# Characterization and Identification of Potential Antioxidant, Antidiabetic, and Antihypertensive Peptides From Hydrolysates of *Tenebrio molitor* Flour and Its Protein Concentrate

**DOI:** 10.1111/1750-3841.70595

**Published:** 2025-09-30

**Authors:** Francielle Miranda de Matos, Ruann Janser Soares de Castro

**Affiliations:** ^1^ Department of Food Science and Nutrition, School of Food Engineering University of Campinas Campinas SP Brazil

**Keywords:** bioactive peptides, mealworm, insects, protein hydrolysis

## Abstract

Insects are recognized as a potential alternative source of proteins and have been studied in recent years as a substrate for enzymatic hydrolysis to obtain bioactive peptides. This study aimed to evaluate the production of hydrolysates with antioxidant, antidiabetic, and antihypertensive properties from mealworm (*Tenebrio molitor*) flour and its protein concentrate. The proteases flavourzyme, alcalase, and neutrase were used for hydrolysis, according to an experimental design of mixtures. The highest antioxidant properties for the mealworm flour were found in the control test, where the substrate remained non‐hydrolyzed. The mealworm protein concentrate hydrolysates treated with Flavourzyme exhibited the highest values for ABTS and DPPH radical scavenging activities. The hydrolysis of mealworm flour and its protein concentrate increased their inhibitory activities against α‐amylase, α‐glucosidase, and angiotensin‐converting enzyme. A significant (*p* < 0.05) and positive correlation was observed between the profiles of protein solubilization (*R* ^2^ > 0.96), free amino groups (*R* ^2^ > 0.94), and antioxidant properties (*R* ^2^ > 0.87) of the hydrolysates obtained from integral flour and of its protein concentrate, indicating that the enzyme action was maintained, despite the differences in substrate composition. The results showed that, in terms of the process, protein concentration is not essential for the production of hydrolysates with bioactive properties. The proteomic analysis identified 38 peptides in the < 3 kDa fraction of the mealworm concentrate protein hydrolyzed with Flavourzyme. These sequences were composed of fractions characterized as bioactive, and in silico analysis predicts some potential bioactive peptides, such as the sequence LPAIL.

## 1 Introduction

It is estimated that by 2050 the world population will be approximately 9.7 billion people (United Nations 2022). This population growth is accompanied by an increase in the demand for food, which has raised concerns about the availability of resources to meet this demand (Pyo et al. 2024). Considering this scenario, many studies have considered the use of insects in human food, especially as an alternative source of protein. This is because insects are rich in protein (35%–77%, on a dry basis), and their production generates less environmental impacts compared to obtaining proteins from traditional sources (Oh and Kim 2024). Compared to conventional livestock farming (cattle, pig, and poultry), insect production requires less space, less water, and emits fewer greenhouse gases; in addition, insects have an efficient conversion of nutrients (Chewaka et al. 2023; Pyo et al. 2024).

The most widely bred and commercialized edible insect is *Tenebrio molitor*, known as the mealworm. The centesimal composition of this insect, on a dry matter basis, is approximately 48.21% protein, 34.21% lipids, 8.54% carbohydrates, 4.01% fiber, and 5.03% ash (Cozmuta et al. 2023). Regarding amino acid composition, mealworm larvae are rich in Glu (5.68%), Asp (3.59%), Ala (3.69%), Ile (3.56%), and Leu (3.41%). Among the indispensable amino acids, Leu, Ile, Val, and Lys are the most abundant, whereas Met and His occur at comparatively low levels (Hong et al. 2020; Ravzanaadii et al. 2012). In addition, *Tenebrio molitor* has a good feed conversion rate (between 3 and 6 kg of feed per kg of live weight) and is considered a safe food according to the European Food Safety Agency (Berraquero‐García et al. 2024; Chewaka et al. 2023). Although *Tenebrio molitor* is one of the most well‐accepted insect species in terms of flavor (Oh and Kim 2024), there is still a great aversion on the part of the population to the use of insects in human food (Pyo et al. 2024), therefore, it is believed that the use of transformed insects as ingredients in food may facilitate consumer acceptance (Chewaka et al. 2023).

Some studies that explore the use of *Tenebrio molitor* as a food ingredient include the evaluation of functional properties of their proteins, such as solubility, water‐ and oil‐holding capacity, and gelation; the production of fortified foods, such as bakery products, extruded “insect‐riched” snacks, and meat products (Gkinali et al. 2022); and the obtaining of peptides with a range of bioactive properties from mealworm proteins (Brai et al. 2022; Yoon et al. 2023; Tan et al. 2022). The production of these bioactive peptides is carried out through enzymatic hydrolysis, and the use of mealworm as a substrate can be in the form of mealworm flour (Gonzalez‐de la Rosa et al. 2024) or as a mealworm protein concentrate (Yoon et al. 2023). Regarding the hydrolysis method, the use of commercial enzymes such as Alcalase (Berraquero‐García et al. 2024) and Flavourzyme (Tan et al. 2022) is reported, as well as the simulation of gastrointestinal digestion (Brai et al. 2022) and the combination of these methods (Gonzalez‐de la Rosa et al. 2024).

Considering the diverse strategies used to obtain bioactive peptides from *Tenebrio molitor* hydrolysates, this study aimed to identify the optimal hydrolysis conditions, in terms of substrate composition and proteases used, to produce mealworm hydrolysates with antioxidant, antidiabetic, and antihypertensive properties. For the hydrolysis, an experimental design of mixtures was employed using the proteases flavourzyme, alcalase, and neutrase, which were applied individually and in binary or ternary combinations. Regarding the substrate, it was used as whole mealworm flour and its protein concentrate in order to verify the hydrolysis profile under these conditions. Additionally, this study aimed to identify the sequence of potentially bioactive peptides and applied these structures in in silico studies to predict their bioactivities.

## 2 Material and methods

### 2.1 Reagents

The reagents 2,4,6‐trinitrobenzenesulfonic acid solution (TNBS), 2,2′‐azino‐di‐(3‐ethylbenzothiazoline)‐6‐sulfonic acid (ABTS), 2,2‐diphenyl‐1‐picrylhydrazyl (DPPH), acid 6‐hydroxy‐2,5,7,8‐tetramethyl‐3,4‐dihydrochromene‐2‐carboxylic (trolox), the proteases Flavourzyme 500 L from *Aspergillus oryzae*, Alcalase 2.4 L from *Bacillus licheniformis*, and Neutrase 0.8 L from *Bacillus amyloliquefaciens*, the enzymes α‐amylase from porcine pancreatin and α‐glucosidase from *Saccharomyces cerevisiae*, and the angiotensin converting enzyme (ACE) were purchased from Sigma‐Aldrich (Brazil). The other reagents used were of analytical grade.

### 2.2 Preparation of Hydrolysis Substrates

The dehydrated mealworms (*Tenebrio molitor*) were purchased at a retail store in the city of São Paulo, Brazil (23°32′59.7″S 46°36′50.0″W). The insects were ground to obtain a mealworm flour. Part of this flour was reserved for enzymatic hydrolysis (MF), and another part was used to produce a protein concentrate. The mealworm protein concentrate (MPC) was obtained according to the method described by de Matos et al. (2021), with slight modifications. The insect flour was dispersed in a 0.2% (m:v) sodium hydroxide solution to raise the pH to 11. The mixture was stirred for 1 h at 25°C, centrifuged at 8000 × g for 20 min, and the supernatant was recovered. The pH of the supernatant was adjusted to 4.5 using a 1 mol L^−1^ HCl solution for isoelectric precipitation. After centrifugation, the protein concentrate was resuspended in distilled water and dehydrated in a forced‐air oven (Model TE394/1, Tecnal, Brazil) at 50°C for 24 h.

### 2.3 Determination of Protease Activity

The proteolytic activity of the commercial proteases was determined following the method described by Amaral and de Castro (2024). In an Eppendorf tube, 0.25 mL of 1% (m:v) azocasein, 0.25 mL of 0.1 mol L^−1^ phosphate buffer (pH 7), and 0.5 mL of commercial protease solution were added. The mixture was incubated at 50°C for 40 min, and the reaction was stopped by adding 0.5 mL of 10% (m:v) trichloroacetic acid solution. The reaction mixtures were centrifuged at 8000 × g for 15 min at 25°C, and 1 mL of the supernatant was transferred to another tube, followed by the addition of 1 mL of 5 mol L^−1^ potassium hydroxide solution. The absorbance was measured at 428 nm. The blank was carried out similarly, but trichloroacetic acid was added before the enzyme solution. One unit of protease activity (U) was defined as the amount of enzyme required to produce a 0.01 difference in the absorbance per minute of reaction between the enzymatic reaction and the blank.

### 2.4 Enzymatic Hydrolysis

For each substrate (mealworm flour and its protein concentrate), 10 hydrolysis assays were performed, according to an experimental design of mixtures (Table 1), in order to determine the most suitable protease or combination of them (independent variables) to obtain hydrolysates with different bioactive properties. The commercial proteases were used individually and in binary or ternary combinations, with each component tested at six levels: 0 (0%), 1/6 (16.67%), 1/3 (33%), 1/2 (50%), 2/3 (66.67%), and 1 (100%). The hydrolysis process was carried out following the method described by de Matos et al. (2022). In Erlenmeyer flasks, 5 g of substrate, 50 mL of 100 mmol L^−1^ sodium phosphate buffer, and 100 U mL^−1^ of proteases were added. The dispersions were incubated at 50°C and kept under agitation at 100 rpm for 2 h. Hydrolysis was stopped by heat treatment at 100°C for 20 min, and the solutions were centrifuged at 8000 × g for 20 min. The collected supernatants were frozen and used in subsequent tests. All experiments were compared with the control test (non‐hydrolyzed substrates).

**TABLE 1:** Simplex centroid mixture design for enzymatic hydrolysis of mealworm flour and its protein concentrate, using different commercial proteases.

	*Proteases*		
Run	Flavourzyme 500 L	Alcalase 2.4 L	Neutrase 0.8 L
H1	1	0	0
H2	0	1	0
H3	0	0	1
H4	1/2	1/2	0
H5	1/2	0	1/2
H6	0	1/2	1/2
H7	2/3	1/6	1/6
H8	1/6	2/3	1/6
H9	1/6	1/6	2/3
H10	1/3	1/3	1/3

The dependent variables (responses) were protein and free amino groups contents, ABTS and DPPH radical scavenging activities, ferric reducing antioxidant power (FRAP), and capacity to inhibit the activity of α‐amylase, α‐glucosidase, and angiotensin‐converting enzymes (ACEs). Linear, quadratic, or cubic equations were employed to define the model for each dependent variable analyzed, as outlined below (Equation 1):

(1) $$Yi=\sum_{i=1}^{q}\beta iXi+\sum \sum_{i\;<\;j}^{q}\beta ijXiXj+\sum \sum_{i\;<\;j\;<\;k}^{q}\sum \beta ijkXiXjXk$$

Where “Yi” denotes the response predicted by the model, “q” represents the number of components in the system, “Xi,Xj,Xk” are the coded independent variables, “βi” refers to the linear regression coefficients for each term's effect, and “βij” and “βijk” represent the interaction effects between binary and ternary mixtures. The coefficient of determination (*R^2^*) and the Fisher test (analysis of variance—ANOVA) were employed to assess the statistical validity of the proposed models. Three additional experiments were performed under the optimal conditions identified through the statistical analysis of the mixture design to validate the models. The experimental values from the validation tests were compared to the predicted values within a 95% confidence interval.

### 2.5 Determination of Protein Content

The protein content of the supernatants collected after hydrolysis was determined by the Lowry method (Hartree 1972), and it was reported as the soluble protein content. In glass tubes, 200 µL of the sample were added to 180 µL of Reagent A (a solution of 0.2% sodium potassium tartrate and 10% sodium carbonate in 0.5 mol L^−1^ NaOH). The mixture was incubated at 50°C for 10 min, followed by the addition of 20 µL of Reagent B (a solution of 1% sodium potassium tartrate and 0.5% copper sulfate pentahydrate in 0.05 mol L^−1^ NaOH), and allowed to stand for 10 min at 25°C. Subsequently, 600 µL of diluted Folin–Ciocalteu reagent (1:15 in water) was added to the tubes, and the mixture was incubated again at 50°C for 10 min. After cooling, absorbance was measured at 660 nm using a Multiskan GO spectrophotometer (Thermo Fisher Scientific, Finland). A standard curve was constructed using ovalbumin solutions (0–0.4 mg/mL), and the results were expressed as mg of protein per mL.

### 2.6 Determination of Free Amino Groups Content

The free amino group content was determined using the TNBS method, following the procedure described by Borges et al. (2023), with slight modifications. The reaction was performed by mixing 200 µL of the sample with 600 µL of 200 mmol L^−1^ sodium phosphate buffer (pH 8.2) and 200 µL of 0.025% TNBS reagent. The mixture was incubated at 50°C for 1 h, and absorbance was measured at 340 nm using a Multiskan GO spectrophotometer (Thermo Fisher Scientific, Finland). A standard curve was generated using L‐leucine solutions (0–20 µg mL^−1^), and the results expressed as mg of L‐leucine equivalents per mL of sample (mg Leu eq. mL^−1^) were converted to a percentage content (%), relative to the total concentration of proteins in the samples, as described in the following equation (Equation 2):

(2) $$\mathrm{Free}\;\mathrm{amino}\;\mathrm{groups}\;\left(\%\right)=\left[\frac{mg\;Leu\;eq.\;mL^{-1}}{mg\;protein\;mL^{-1}}\right]\times 100$$

### 2.7 Antioxidant Properties

The antioxidant properties of the samples were measured in terms of ABTS radical scavenging activity (Re et al. 1999), DPPH radical scavenging activity (Brand‐Williams, Cuvelier, and Berset 1995), and FRAP (Benzie and Strain 1996), according to the methods described by de Matos et al. (2024). The determinations were performed in triplicate, and the absorbances were measured in a microplate reader (Multiskan GO, Thermo Fisher Scientific, Finland). Trolox was used as the reference standard, and results were expressed in µmol of Trolox equivalent per gram of protein (µmol TEq g^−1^).

### 2.8 Antidiabetic Properties

The antidiabetic properties of the samples were measured in terms of the capacity to inhibit the activity of the enzymes α‐amylase from porcine pancreatin and α‐glucosidase from *Saccharomyces cerevisiae*, according to the methods described by de Matos, Rasera, and de Castro (2024). For both analyses, a control assay, corresponding to the reaction in the absence of an inhibitor, was prepared by replacing the sample in the reaction mixture with 100 mmol L^−1^ phosphate buffer (pH 6.9). The inhibition of α‐amylase and α‐glucosidase activities was calculated according to Equation 3, as follows:

(3) $$\mathrm{Inhibition}\;\left(\%\right)=\left[\frac{\Delta Abs.Control-\Delta Abs.Sample}{\Delta Abs.Control}\right]\times 100$$

### 2.9 ACE Inhibitory Activity

The potential antihypertensive activity of the samples was measured based on the capacity to inhibit the activity of the ACE, using the method described by de Matos et al. (2024). For this, ACE enzyme from rabbit lung and the substrate hippuryl‐histidyl‐leucine were used. The enzyme activity was determined in terms of the concentration of hippuric acid, which was measured at 228 nm using the Multiskan GO spectrophotometer (Thermo Fisher Scientific, Finland). A control assay, corresponding to the reaction in the absence of an inhibitor, was prepared by replacing the sample in the reaction mixture with buffer, and the blank samples were prepared by replacing the enzyme and substrate with boric acid‐borax buffer (pH 8.3). ACE activity inhibition was calculated according to Equation 4, as follows:

(4) $$\mathrm{Inhibition}\;\left(\%\right)=\left[\frac{\Delta Abs.Control-\;\Delta Abs.Sample}{\Delta Abs.Control}\right]\times 100$$

### 2.10 Ultrafiltration Fractionation

The samples indicated by the validated statistical models with the highest potential bioactive properties were fractionated. Ultrafiltration membranes with molecular mass cutoffs of 30, 10, 5, and 3 kDa (Merck Millipore, USA) were used. The protein content of the fractions was determined by the Lowry method (Hartree 1972), and their potential bioactive property was measured to identify the fraction with the highest bioactivity.

### 2.11 Mass Spectrometry Analysis

#### 2.11.1 Sample Preparation

The fraction with a molecular mass below 3 kDa of the sample MPC‐H1 (mealworm protein concentrate hydrolyzed by Flavourzyme) was dried in a vacuum evaporator (SpeedVac, Thermo Scientific, Waltham, USA). The sample was reconstituted in 220 µL of 0.1% trifluoroacetic acid (TFA) in water and cleaned up with a C18 micro‐pipette tip (Merck, Darmstadt, Germany). The eluate was dried again and reconstituted in a 30 µL solution of 97:3 water acetonitrile (ACN) 0.1% formic acid (FA). Immediately before desalting, the pH of the sample was adjusted to <4 with 0.1% TFA solution.

#### 2.11.2 Desalting

For the desalting, a C18 micro‐pipette tip (Merck, Darmstadt, Germany) was conditioned with 100% ACN and washed twice with the 0.1% TFA equilibration solution. The sample was loaded onto the tip in 7 to 10 cycles. The tip was washed twice with a 0.1% TFA solution, and the peptides were eluted using a 0.1% FA in 60% ACN solution. The eluate was dried in the vacuum evaporator and reconstituted in a 97:3 water 0.1% FA solution.

#### 2.11.3 Micro Liquid Chromatography Mass Spectrometry

The recovered peptides were analyzed in a system composed of a microACQUITY UPLC coupled with a Xevo G2‐XS Q‐ToF mass spectrometer, and equipped with a LockSpray ion source (Waters, USA). Firstly, the columns were equilibrated with 93% mobile phase A (0.1% FA in water) and 7% mobile phase B (0.1% FA in ACN). The temperature of the column was set to 40°C, and 10 µL of the sample was injected into the system. The peptides were loaded on a trap column (ACQUITY UPLC Symmetry C18, 5 µm particle size, 300 µm × 25 mm) at a flow rate of 15 µL min^−1^ for 4 min. Then, the peptides were separated on an analytical column (ACQUITY UPLC HSS T3, 1.8 µm particle size, 300 µm × 150 mm) at 5 µL min^−1^ flow rate with a gradient of 7% to 40% of mobile phase B over 82 min, followed by a 6 min rinse of 85% and 24 min for equilibration in 7%. Data dependent acquisition mode (DDA) was carried out by operating the Xevo G2‐XS QTof MS in the positive mode, and applying the MS and MS/MS functions over 0.5 s intervals with 6 V low energy and 15–45 V high energy collision. The Leucine‐Enkephalin (556.2771 Da) was the internal mass calibrant. The MS Survey Switching Threshold was 10.000, and peptide signal data were collected between 100 and2000 m/z values, a range that covers the mass spectra of most peptides.

#### 2.11.4 Proteomics Data Processing

Raw data files were processed using the ProteinLynx Global Server search engine (PLGS, version 3.0.3, Waters) against the UniProtKB database (Swiss‐Prot, Coleoptera, Taxon ID: 7041, release August 2024).

Processing parameters for the Electrospray Survey module were set to ensure accurate mass measurements and high‐quality spectra. Lock mass correction was applied with 3 Lock Spray scans and a tolerance of 0.5 Da. Noise reduction was performed using adaptive background subtraction (threshold 35%, polynomial 5). Smoothing was applied using the Savitzky‐Golay algorithm with 2 iterations and a 3‐channel window. Deisotoping was performed using the “Fast” method with 30 interactions, a threshold of 3%, and a minimum peak width of 4 channels. Centroiding was applied using Top 80% mode, with TOF resolution set to 23,000 and an NP multiplier of 1. These settings were used for all spectra to ensure reproducibility of peptide and protein identifications.

Peptide and protein identifications were performed with a minimum of three fragment ion matches per peptide and five per protein, requiring at least one peptide per protein. The following parameters were applied: peptide tolerance of 100 ppm; estimated calibration error of 0.005 Da; molecular weight range of 0–200,000 Da; pI range of 0–14; no specific enzyme cleavage; no more than one missed cleavage; carbamidomethylation on C (+57.02 Da) and oxidation on M (+15.99 Da) as variable modifications; and a false discovery rate (FDR) threshold of 1%.

#### 2.11.5 In Silico Prediction of Bioactivities

The BIOPEP‐UWM database (available at http://www.uwm.edu.pl/biochemia/index.php/en/biopep; accessed on October 26, 2024) was used to verify the occurrence of biological activities in the identified peptides or their fragments. This platform was also used to convert the peptides into SMILES sequences. The peptides were evaluated for their antioxidant potential with the AnOxPePred platform (available at https://services.healthtech.dtu.dk/services/AnOxPePred‐1.0/; accessed on October 27, 2024). Peptides with scores above 0.5 were considered potential free radical scavengers (FRS) or ion chelators (CHEL). The identification of potential ACE inhibitory peptides was analyzed using the AHTpin server (available at https://webs.iiitd.edu.in/raghava/ahtpin/index.php; accessed on October 27, 2024), with the support vector machine (SVM) threshold set to >1. The SMILES sequences of the peptides were analyzed on the SwissTargetPrediction platform to identify sequences with potential inhibitory activities related to antidiabetic and antihypertensive properties. The PDB structures of the peptides of interest were generated on the PEP‐FOLD4 platform (available at https://bioserv.rpbs.univ‐paris‐diderot.fr/services/PEP‐FOLD4/; accessed on October 28, 2024). In silico molecular docking was performed on the PepSite2 server (available at http://pepsite2.russelllab.org/; accessed on October 28, 2024). The PDB structures of the inhibited enzymes were obtained from the RCSB protein data bank (PDB). The molecular weight, isoelectric point, and net charge at pH 7 of the peptides were estimated using the Peptide2 software (https://www.peptide2.com/peptide_protein_sequence_analysis.php; accessed on September 01, 2025), whereas their solubility was predicted with the PepCalc software (https://pepcalc.com/; accessed on September 01, 2025). The resulting physicochemical properties were summarized in Table S4 (Supplementary material 4).

### 2.12 Statistical Analyses

The results are presented as the arithmetic mean ± standard deviation (n = 3). ANOVA followed by Tukey's test was performed using Minitab 19 software (State College, PA, USA). The means were considered statistically different when the *p*‐value was < 0.05. The Pearson correlation coefficient was applied to evaluate the strength of linear relationships between certain responses, with values ranging from −1 to 1 (indicating perfect negative and positive correlation, respectively). Correlations were considered significant when *p* < 0.05.

## 3 Results and Discussion

### 3.1 Soluble Protein and Free Amino Groups Contents

The highest soluble protein contents were detected for the substrates hydrolyzed using the protease Neutrase (Table 2). For the hydrolysate of mealworm flour (MF‐H3), the increase in protein content was approximately 221% compared to the control (non‐hydrolyzed sample). For the mealworm protein concentrate hydrolysate (MPC‐H3), this increase was of approximately 211% (Table 2). This determination was important for the quantification of bioactive properties, given that these are expressed as a function of the protein concentration in the samples. By performing the Pearson correlation test, it was possible to observe a high, positive, and significant correlation (0.964, *p* < 0.001) between the soluble protein profiles of the two groups of samples (MF and MPC). This showed that the profiles of action of the proteases were maintained despite the differences between the substrates compositions.

**TABLE 2:** Protein content and percentage of free amino groups of mealworm flour (MF) hydrolysates and mealworm protein concentrate (MPC) hydrolysates.

	Mealworm flour (MF)		Mealworm protein concentrate (MPC)	
Run	Protein content (mg mL^−1^)	Free amino groups (%)	Protein content (mg mL^−1^)	Free amino groups (%)
**Control**	17.78 ± 0.21^g^	11.50 ± 0.13^f^	35.62 ± 2.12^f^	1.21 ± 0.14^j^
**H1**	36.51 ± 0.51^f^	26.77 ± 1.31^a^	60.14 ± 1.57^e^	21.95 ± 0.43^a^
**H2**	48.41 ± 0.97^c^	7.82 ± 0.07^h^	87.49 ± 1.47^c^	3.56 ± 0.07^i^
**H3**	57.07 ± 0.70^a^	9.15 ± 0.07^g^	110.61 ± 3.01^a^	5.55 ± 0.10^h^
**H4**	41.41 ± 0.19^e^	22.41 ± 0.09^c^	79.66 ± 1.07^d^	15.71 ± 0.11^c^
**H5**	42.06 ± 0.14^e^	25.43 ± 0.54^b^	84.99 ± 3.76^c, d^	15.94 ± 0.11^c^
**H6**	56.51 ± 0.62^a^	9.59 ± 0.12^g^	102.71 ± 0.80^b^	6.10 ± 0.04^g^
**H7**	42.46 ± 0.21^e^	22.49 ± 0.21^c^	82.42 ± 2.28^c, d^	18.29 ± 0.20^b^
**H8**	49.70 ± 1.70^c^	17.56 ± 0.12^e^	102.58 ± 1.86^b^	10.93 ± 0.14^f^
**H9**	52.66 ± 0.28^b^	17.11 ± 0.17^e^	100.46 ± 0.67^b^	11.48 ± 0.05^e^
**H10**	45.94 ± 0.14^d^	20.33 ± 0.03^d^	98.15 ± 0.00^b^	14.06 ± 0.10^d^

The results were expressed as the mean (n = 3) ±standard deviation (SD). The values with different letters within the same column are significantly different (*p* < 0.05).

About the free amino groups content, the greatest modifications compared to the control samples were obtained through hydrolysis using flavourzyme. For the hydrolyzed mealworm flour (MF‐H1), the free amino group content increased by approximately 2.3‐fold compared to the control sample (MF‐C). For the hydrolyzed mealworm protein concentrate (MPC‐H1), this increase was approximately 18‐fold compared to the control sample (MPC‐C). The Pearson coefficient between the free amino groups content of hydrolysates of MF and MPC was 0.944 (*p* < 0.001), indicating a strong positive correlation, while confirming that both the hydrolysis profile and enzymatic activity remained consistent regardless of substrate type.

The protease neutrase from *Bacillus amyloliquefaciens* is a neutral, zinc metallo endo‐protease (Mostafavi et al. 2023) that specifically targets bonds involving Leu and Phe residues (Bing et al. 2024). According to Min et al. (2024), the hydrolysis of yeast protein with neutrase generated hydrolysates with higher solubility, when compared with the application of alcalase, flavourzyme, or prozyme. The cleavage by neutrase generated protein hydrolysates with more uniform particle size distribution, releases polar functional groups due to cleavage of peptide bonds, and exposes hydrophilic structures that were previously hidden in the native structure, increasing protein solubilities (Min et al. 2024). These findings are in line with the results obtained in the present study.

On the other hand, the protease flavourzyme from *Aspergillus oryzae* is a complex mixture of endo‐ and exopeptidases, that have a broader specificity to release very small peptides from lineal chains (amino‐peptidase) and free amino acids (Rivero‐Pino, Espejo‐Carpio, and Guadix 2020; Xu et al. 2020). This protease exhibited high specificity for Lys, Gln, and Leu residues, and the release of free amino acids occurs mainly from the C‐terminal (Wang et al. 2024; Sinthusamran et al. 2020). The specificity of flavourzyme explains the fact that its use in the hydrolysis of mealworm resulted in hydrolysates with the highest contents of free amino groups. Similar results were observed by Min et al. (2024), in which the hydrolysis of yeast protein with flavourzyme generated hydrolysates with a higher total amino acid content, even though the highest soluble protein content was obtained by using neutrase.

**FIGURE 1:**
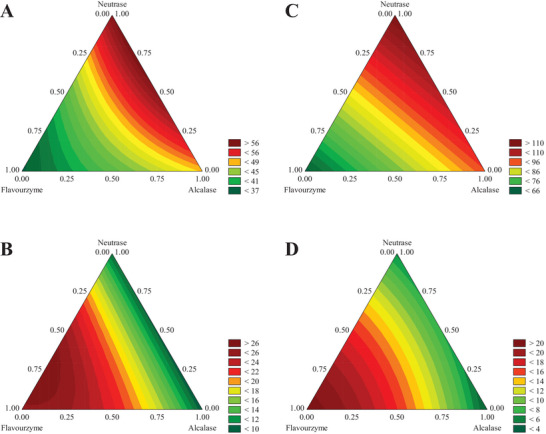
Mixture‐contour plots for protein content (mg mL^−1^) (A and C) and free amino groups (%) (B and D) of mealworms hydrolysates using a statistical mixture design. The graphics A and B correspond to the flour hydrolysates, and the graphics C and D correspond to the protein concentrate hydrolysates.

The ANOVA showed that 98% and 87% of the total variation (R^2^) of the protein content of MF and MPC hydrolysates, respectively, could be explained by mathematical models (Supplementary material 1). For the free amino groups content, the generated models can explain 95% and 98% of variations (R^2^) in this quantification for MF and MPC hydrolysates, respectively. The values for the F‐test were higher than the F‐tabulated at *p*‐value < 0.05. Contour plots (Figure 1) were generated using the significant parameters (*p* < 0.10) for each response, evidencing the main and interaction effects of the commercial proteases in the production of protein hydrolysates with high soluble protein and free amino group contents. The angles of the triangles correspond to the responses for the isolated use of proteases, the midpoints on the sides are related to the use of binary mixtures, and the central points refer to the ternary mixtures. Figures 1A and 1C show that hydrolysis with a binary combination of neutrase and alcalase—with a predominance of neutrase—generates the hydrolysates with the highest protein content for both substrates. On the other hand, Figures 1B and 1D confirm that the enzyme flavourzyme generates the best responses for the content of free amino groups for both substrates.

### 3.2 Bioactive Properties

#### 3.2.1 Antioxidant Properties

Regarding the antioxidant properties, for the mealworm flour, it is possible to observe that all samples exhibited the activities of interest (Table 3). It is important to note that for the three methods, the non‐hydrolyzed samples exhibited the highest antioxidant activities. There are two hypotheses for this behavior: (i) the final values were adjusted based on the soluble protein content. Since the non‐hydrolyzed samples have a lower protein concentration (Table 2), their adjusted antioxidant activity values are higher; and (ii) enzymatic hydrolysis of proteins may release peptides and also result in greater release of lipids from the flour. Given that *Tenebrio* larvae contain approximately 34% lipids in their dry mass, with about 80% of these being unsaturated fatty acids (Cozmuta et al. 2023), their oxidation may reduce the antioxidant activity of the hydrolyzed samples.

**TABLE 3:** Antioxidant properties of the hydrolysates from mealworm flour and its protein concentrate obtained by enzymatic hydrolysis using the statistical mixture design and different proteases and their combinations.

Antioxidant properties of mealworm flour and its protein concentrate (µmol TEq g^−1^)						
	ABTS		DPPH		FRAP	
Run	Flour	Concentrate	Flour	Concentrate	Flour	Concentrate
**Control**	284.79 ± 11.97^a^	153.28 ± 10.51^g^	50.33 ± 4.73^a^	6.71 ± 1.49^b, c, d^	121.18 ± 3.98^a^	21.26 ± 1.09^a^
**H1**	234.51 ± 5.24^b^	373.51 ± 9.76^a^	45.09 ± 2.11^a, b^	11.04 ± 1.65^a^	67.01 ± 1.89^b^	18.23 ± 0.26^b^
**H2**	179.86 ± 3.90^e, f^	295.96 ± 6.35^c, d, e^	27.33 ± 0.73^f, g^	5.99 ± 1.03^b, c, d^	46.41 ± 1.59^g, h^	10.83 ± 0.77^f, g^
**H3**	168.73 ± 4.23^f^	267.07 ± 9.09^f^	26.72 ± 0.84^f, g^	5.43 ± 0.11^c, d^	42.94 ± 1.70^h^	9.65 ± 0.89^g^
**H4**	215.08 ± 2.38^c^	325.13 ± 5.39^b^	38.75 ± 3.26^c^	7.37 ± 0.97^b, c, d^	60.68 ± 1.93^c^	13.78 ± 1.67^d, e^
**H5**	212.82 ± 2.88^c^	298.48 ± 5.12^c, d^	32.49 ± 3.50^d, e, f^	7.41 ± 1.69^b, c, d^	59.15 ± 1.57^c, d^	14.92 ± 0.77^c, d^
**H6**	166.95 ± 2.95^f^	287.83 ± 8.99^d, e^	24.30 ± 1.22^g^	4.63 ± 0.64^d^	43.96 ± 1.78^g, h^	10.90 ± 0.69^f, g^
**H7**	197.60 ± 2.68^d^	308.95 ± 6.89^b, c^	36.38 ± 1.34^c, d^	8.12 ± 1.00^a, b, c^	57.33 ± 1.04^c, d^	16.19 ± 0.64^b, c^
**H8**	176.63 ± 4.22^e, f^	279.99 ± 5.02^e, f^	34.16 ± 2.44^c, d, e^	7.05 ± 1.37^b, c, d^	51.42 ± 1.39^e, f^	11.71 ± 0.35^f^
**H9**	182.75 ± 6.24^e^	278.82 ± 5.42^e, f^	29.43 ± 1.08^e, f, g^	7.11 ± 1.83^b, c, d^	47.65 ± 0.89^f, g^	12.81 ± 0.76^e, f^
**H10**	184.07 ± 4.44^e^	286.06 ± 2.65^d, e^	39.21 ± 2.33^b, c^	8.82 ± 1.04^a, b^	55.47 ± 0.89^d, e^	14.05 ± 0.32^d, e^

The antioxidant properties were expressed in µmol of Trolox equivalent per gram of protein hydrolysate (µmol TEq g^−1^) and presented as the mean (n = 3) ± SD. The values with different letters within the same column are significantly different (*p* < 0.05). The relative antioxidant activity (RAA) (%) was calculated as the percentual variation of the antioxidant activity (µmol TEq g^−1^) obtained for the hydrolyzed samples in comparison with their respective control assays (non‐hydrolyzed protein).

For the mealworm protein concentrate, the best antioxidant properties (ABTS and DPPH) were achieved using flavourzyme (MPC‐H1), while the highest FRAP activity was obtained for the non‐hydrolyzed sample (MPC‐C) (Table 3). Analyzing the data regarding the action of the enzymes (samples H1 to H10), it is possible to observe that flavourzyme is the best enzyme to obtain MF and MPC hydrolysates with antioxidant properties. Despite the compositional differences between the substrates, the action profile of the commercial proteases is maintained, so that there is a high, positive, and significant correlation (*p* < 0.05) between the same antioxidant activity for the two different substrates (Pearson correlation coefficient of 0.887 for ABTS, 0.940 for DPPH, and 0.922 for FRAP).

In a study by Botella‐Martínez et al. (2021), the chemical composition and the antioxidant properties of defatted mealworm flour were determined. The protein content of the sample was approximately 64%, and the antioxidant activities were 3.12, 0.76, and 3.08 mg Trolox g^−1^ flour for the ABTS, DPPH, and FRAP assays, respectively. These values showed a moderate correlation with the protein content of the mealworm flour, but a strong correlation with the composition of total phenolic and tannin contents of the sample.

The antioxidant properties of the hydrolysates obtained in our study are comparable to the activities obtained for other insect hydrolysates. Proteins from *Alphitobius diaperinus* hydrolyzed with Alcalase and Corolase showed ABTS radical scavenging activity of 95.0 and 95.7 µmol TE g^−1^, respectively (Sousa, Borges, and Pintado 2020). Protein hydrolysates of cricket (*Acheta domesticus*) obtained using Alcalase were fractionated by size exclusion chromatography. The fraction with the highest properties showed antioxidant activities of 215.9, 360.9, and 201.8 mg TE g^−1^, measured by ABTS, DPPH, and FRAP assays (Summart et al. 2024). Establishing correlations in studies on mealworm hydrolysate production is challenging due to differences in activity units used; however, some studies highlight the importance of the Flavourzyme enzyme in enhancing the antioxidant properties of mealworm protein hydrolysates (Gonzalez‐de la Rosa et al. 2024; Rivero Pino et al. 2020).

According to the literature, antioxidant peptides tend to have a low molecular weight, generally below 1000 Da, allowing their amino acids to be more exposed for reaction. Additionally, they tend to be composed of hydrophobic, aromatic amino acids or those with an imidazole ring. Finally, the antioxidant activity of peptides relies not only on their composition but also on the specific arrangement of amino acids and the overall configuration of the peptides (de Matos and de Castro 2021; Zou et al. 2016). Considering these characteristics and the action profile of Flavourzyme, it is reasonable that the hydrolysates obtained from this protease exhibited the best antioxidant properties.

For the antioxidant properties, the ANOVA showed that 80% to 97% of the total variation in the activities of both substrates could be explained by mathematical models, and all values for the F‐test were higher than the F‐tabulated at *p*‐value < 0.05 (Supplementary material 1). The contour plots generated using the significant parameters (*p* < 0.10) for each response confirmed that flavourzyme, when applied individually, generates the best responses for the antioxidant activities of both substrates (Supplementary material 2).

#### 3.2.2 Antidiabetic and Antihypertensive Properties

Regarding antidiabetic properties, the highest inhibition rates of α‐amylase activity were 19.88% for the mealworm flour and 27.89% for the protein concentrate, both were hydrolyzed by the enzyme alcalase (runs MF‐H2 and MPC‐H2) (Table 4). For the α‐glucosidase, the highest inhibition rates were 15.36% for the mealworm flour hydrolyzed with the combined use of the three proteases and predominance of alcalase (MF‐H8), and 17.36% for the mealworm protein concentrate hydrolyzed also with the ternary combination of enzymes, but with predominance of flavourzyme (MPC‐H7).

**TABLE 4:** Potential antidiabetic and antihypertensive properties of the hydrolysates from mealworm obtained by enzymatic hydrolysis using the statistical mixture design and different proteases and their combinations.

	Mealworm flour			Mealworm protein concentrate		
Run	α‐amylase inhibitory activity (%)	α‐glucosidase inhibitory activity (%)	ACE inhibition (%)	α‐amylase inhibitory activity (%)	α‐glucosidase inhibitory activity (%)	ACE inhibition (%)
**Control**	−2.84 ± 0.21^h^	13.32 ± 0.98^b, c^	47.37 ± 0.92^b^	26.18 ± 0.33^a, b^	13.56 ± 0.20^c, d^	−23.58 ± 3.29^f^
**H1**	5.02 ± 0.20^e^	5.11 ± 0.70^f^	53.20 ± 3.08^a^	26.39 ± 0.74^a, b^	10.61 ± 1.27^e^	44.84 ± 1.75^c, d^
**H2**	19.88 ± 0.11^a^	9.55 ± 0.86^e^	36.79 ± 2.19^d, e, f^	27.87 ± 0.80^a^	10.54 ± 0.91^e^	41.28 ± 0.75^d^
**H3**	−8.43 ± 0.29^i^	11.00 ± 0.06^d, e^	56.89 ± 1.70^a^	24.44 ± 0.24^b, c^	12.45 ± 0.60^d, e^	46.50 ± 2.08^b, c, d^
**H4**	−0.63 ± 0.01^g^	14.50 ± 0.17^a, b^	38.11 ± 0.79^d, e^	20.96 ± 1.03^e^	15.93 ± 0.66^a, b^	42.47 ± 1.19^c, d^
**H5**	0.52 ± 0.39^f^	12.96 ± 0.29^b, c^	40.98 ± 1.81^c, d^	21.19 ± 0.26^d, e^	14.94 ± 0.50^b, c^	41.56 ± 1.20^d^
**H6**	6.94 ± 0.11^d^	14.69 ± 0.33^a, b^	45.85 ± 3.02^b, c^	23.26 ± 0.15^c, d^	12.06 ± 1.23^d, e^	54.60 ± 2.13^a^
**H7**	6.47 ± 0.21^d^	15.20 ± 0.30^a^	38.40 ± 2.00^d, e^	21.63 ± 1.44^d, e^	17.36 ± 0.74^a^	29.02 ± 0.23^e^
**H8**	5.02 ± 0.20^e^	15.36 ± 0.55^a^	31.60 ± 1.18^f^	25.28 ± 0.36^b, c^	13.89 ± 0.08^b, c, d^	48.10 ± 2.66^b, c^
**H9**	14.75 ± 0.34^b^	11.75 ± 1.10^c, d^	57.04 ± 2.66^a^	9.74 ± 0.52^f^	13.66 ± 0.38^c, d^	50.99 ± 3.18^a, b^
**H10**	12.95 ± 0.14^c^	9.72 ± 0.31^e^	35.05 ± 0.76^e, f^	25.60 ± 0.89^b^	11.38 ± 0.50^e^	47.82 ± 2.50^b, c^

Results are presented as the mean (n = 3) ± SD, and those with different letters within the same column are significantly different (p < 0.05). The samples were analyzed at a concentration of 5 mg ptn mL^−1^ for α‐amylase and α‐glucosidase and 1 mg ptn mL^−1^ for ACE.

Fractions (<0.5 kDa) from cricket (*Gryllodes sigillatus*) hydrolyzed with alcalase and subjected to simulated gastrointestinal digestion were able to inhibit 50% of α‐amylase and α‐glucosidase activities at low concentrations (18.5 and 13.9 µg/mL, respectively) (Hall et al. 2020). Additionally, mealworm protein hydrolysates exhibiting 90% α‐glucosidase inhibitory activity were produced through a 7 h hydrolysis process utilizing the enzyme trypsin. The hydrolysis time was reduced to approximately 2.5 h by applying a 15 min sonication pre‐treatment to the mealworm meal, followed by sequential hydrolysis with Alcalase and trypsin. Using this method, the ultrasound pre‐treatment, combined with 1 h of hydrolysis with Alcalase and 1.5 h with trypsin, generated hydrolysates with nearly 100% α‐glucosidase inhibitory activity (Rivero‐Pino et al. 2020).

Peptides with α‐amylase inhibition activity can act competitively, binding to residues Asp197, Glu233, and Asp300 of the enzyme, which catalyze the hydrolysis of glycosidic bonds. On the other hand, the binding/interaction of peptides in other regions, such as residues Trp58, Tyr62, His101, Pro163, Tyr258, and Ala307, can lead to non‐competitive inhibition of α‐amylase, promoting changes in its structural conformation (Yan et al. 2019). According to Fan et al. (2024), hydrophilic amino acids, such as Cys and Arg, hydrophobic amino acids, and aromatic amino acids tend to interact with human α‐amylase, inhibiting the enzyme activity mainly through hydrophobic interactions and hydrogen bonds. Therefore, it is desirable that these amino acid residues be present and accessible in the peptides of interest.

Some studies indicate that α‐glucosidase inhibitor peptides are typically composed of 3–6 amino acids, with hydroxylated or basic charge side chain amino acids at the N‐terminal end of the peptide. Additionally, the presence of proline within the chain and alanine or methionine at the C‐terminal is desirable (Ibrahim et al. 2018). Regarding the structure of the enzyme, α‐glucosidase inhibitors should bind mainly between residues 358–720 of the enzyme, of which Arg, Trp, Cys, and Asp play an important role. Inhibition can occur by binding/interactions of the inhibitors with the active site and/or through interactions that induce conformational changes in the enzyme (Zheng et al. 2024).

Concerning the antihypertensive property, the mealworm flour hydrolysate obtained by the ternary combination of proteases, with a predominance of Neutrase (MF‐H9), showed the highest ACE inhibition activity (57.04%). For the hydrolysate obtained from the protein concentrate, the highest ACE inhibition activity (54.69%) was achieved using the binary combination of Alcalase and Neutrase (MPC‐H6) (Table 4). Similar results were observed in a study by Mendonza‐Salazar et al. (2021), in which hydrolysates of mealworm flour were obtained through fermentation. The best ACE inhibitory activity was found in the protein fraction with a molecular weight below 3 kDa from the sample fermented for 24 h by *Lactococcus lactis* NRRL B‐50572, reaching 50% of the ACE inhibition with the sample at a concentration of 0.97 mg mL^−1^.

According to the literature, peptides with ACE inhibitory activity generally have short chains, composed of 2 to 12 amino acids. Despite this, peptides with a molecular weight below 400 Da (dipeptides and tripeptides) are more common, as they have a greater capacity to fit into the ACE active site. Some common amino acids at the N‐terminal of peptides with antihypertensive activity include leucine, valine, isoleucine, alanine, glycine, tyrosine, and phenylalanine. On the other hand, proline, tyrosine, phenylalanine, isoleucine, and leucine are commonly found at the C‐terminal (Chen et al. 2021; Ding et al. 2023). ACE inhibition can be competitive or noncompetitive. In competitive inhibition, peptides bind to the enzyme's active site via hydrogen bonds. ACE has three active site pockets, which include residues Ala354, Glu384, and Tyr523 (S1), Gln281, His353, Lys511, His513, and Tyr520 (S2), and Glu162 (S1′) (Lu et al. 2021).

It was not possible to generate significant models (*p* < 0.05) that could explain the variations in the inhibition activities of α‐amylase, α‐glucosidase, and ACE. No high or significant correlation was observed between these activities in the mealworm hydrolysates obtained from flour and its protein concentrate. The mathematical models obtained for the antioxidant properties were validated (Supplementary material 3). For this, the mealworm hydrolysates produced using flavourzyme alone were again produced in triplicate, and their antioxidant activities were re‐evaluated and compared with the values predicted by the mathematical model.

### 3.3 Fractionation by Ultrafiltration

**FIGURE 2:**
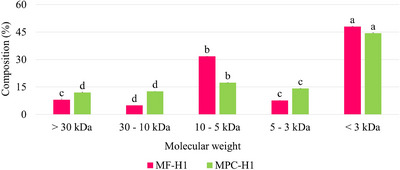
Molecular weight distribution of mealworm flour (MF‐H1) and its protein concentrate (MPC‐H1) hydrolyzed using flavourzyme. Different letters between the fractions of a same sample indicate significant difference (*p* < 0.05).

After validation, the hydrolyzed substrates with Flavourzyme were fractionated using ultrafiltration membranes. These samples showed the best results for most of the bioactivities evaluated. The mealworm flour hydrolysate was mainly composed of peptides with molecular weight (MW) less than 3 kDa (47.91%), followed by molecules with MW between 10 and 5 kDa (31.68%). For the mealworm protein concentrate hydrolysate, 44.28% and 17.25% of their content were of MW less than 3 kDa and between 10 and 5 kDa, respectively (Figure 2).

**FIGURE 3:**
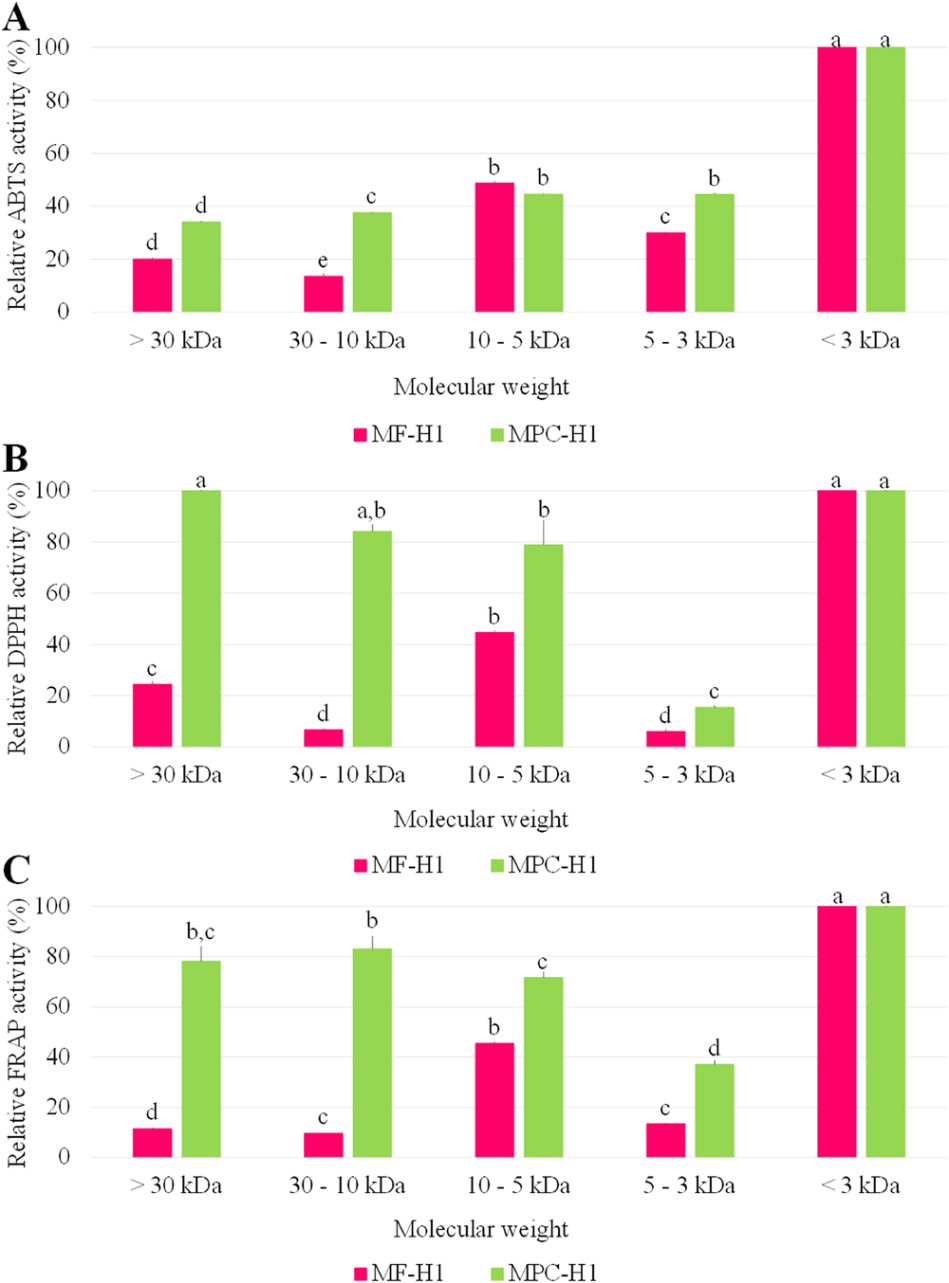
Relative antioxidant activities of the fractions of mealworm flour (MF‐H1) and its protein concentrate (MPC‐H1) hydrolyzed using Flavourzyme.

The antioxidant properties of these fractions were determined and expressed as relative antioxidant activity (%). The fraction with the highest activity was considered 100% active, and the other values were expressed as functions of this. For the three antioxidant analyses, the higher activities were obtained in the fractions with MW less than 3 kDa (Figure 3). Fractions of the mealworm protein concentrate hydrolysate (MPC‐H1) with a molecular weight greater than 5 kDa also showed relative activities greater than 70% for DPPH and FRAP methods.

### 3.4 Peptides Identification and in Silico Bioactive Potential Prediction

Considering the following observations: (a) among the bioactivities studied, mathematical models were generated only for the antioxidant activities; (b) the condition that produced the highest activities was achieved using the enzyme flavourzyme; (c) this condition also generated hydrolysates with the highest degree of hydrolysis; (d) the fractions with the highest activities had a molecular weight below 3 kDa, and e) the protein composition of the protein concentrate does not differ from the protein composition of the mealworm flour: the molecular weight fraction below 3 kDa from the MPC‐H1 sample was purified, and its peptides were identified by data‐dependent (DDA) mass spectrometry. The MS/MS spectra generated and searched in the Coleoptera order database (only reviewed entries) on UniProt enabled the identification of 38 peptide sequences (Table 5). These peptides consisted of 5–16 amino acid residues, with MW ranging from 525 to 1546. All identified peptides exhibited regions similar to bioactive peptides reported on the BIOPEP platform (bold sequences of the peptides).

**TABLE 5:** Potential bioactive peptides identified from fraction <3 kDa of mealworm protein concentrate hydrolyzed with flavourzyme (MPC‐H1).

Peptide sequence	Mass (Da)	RT (min)	BIOPEP ID	Inhibitor
AP**IAY**	533.29	15.5	10647	Antioxidant
RPQVDLE**TY**	1119.56	38.3	8219	Antioxidant
APLAYSGGY**LH**	1147.57	22.7	3305	Antioxidant
DVQDG**LT**GDSKN	1247.56	8.0	10749	Antioxidant
Y**AAAPVAVAK**	959.54	11.2	9831	α‐glucosidase
**AD**EYDPHPQY	1233.49	11.2	9695	α‐glucosidase
AMKNFGMK**PE**E	1296.58	8.2	9694	α‐glucosidase
MKTQ**DP**	734.33	10.6	8767	DPP‐IV
**IPA**II	525.35	34.8	3507	ACE
AP**LAY**	533.29	15.5	3558	ACE
LPA**IL**	525.35	34.8	9079	ACE
IEP**IF**	617.34	42.5	7593	ACE
ITV**PLP**	638.40	21.7	2664	ACE
GG**YGGY**	572.22	9.4	3493	ACE
**GL**LEGLD	715.38	33.2	7599	ACE
QAAP**VAV**	654.37	15.5	7635	ACE
**AAP**VAVAK	725.44	11.3	3375	ACE
GLIGAP**IA**	710.43	31.9	7562	ACE
APLAAP**AI**	722.43	22.1	8193	ACE
APIAAP**IA**	722.43	22.4	7562	ACE
**AP**IAAPLA	722.43	22.4	7584	ACE
GLGAPA**LG**	654.37	21.0	7619	ACE
**GL**IGAPAVA	767.45	30.0	7599	ACE
**GL**LGAPAVA	767.45	30.0	7599	ACE
GLIGAPI**AAP**	878.52	35.8	3375	ACE
AVAAP**VAV**AK	895.55	11.2	7635	ACE
DIRVSNP**GVR**	1111.61	9.7	9727	ACE
**AA**YAAPVAHA	940.48	10.2	7590	ACE
**LG**GNQAVSHY	1044.50	8.7	7619	ACE
VATYAAAP**VAV**	1031.57	17.8	7635	ACE
**AA**VAAPVAVAK	966.59	12.4	7590	ACE
DIRVSNPG**VRF**	1258.68	24.6	10692	ACE
SLGGNQAVS**HY**	1131.53	10.2	3494	ACE
**GGY**GSGLGIAR	1006.52	16.7	3515	ACE
DIQ**DGL**TGDSKN	1261.58	8.0	9056	ACE
APLAYGAPVA**KY**	1219.66	23.5	7691	ACE
ELHGDSGKGGS**GEP**	1325.58	8.0	7554	ACE
**AP**YAVHAPAVGASHQ	1545.77	13.7	3375	ACE

ACE: Angiotensin I‐converting enzyme inhibitory activity; BIOPEP ID and inhibition activity correspond to the sequences in bold; DPP‐IV: dipeptidyl peptidase‐IV inhibitory activity.

According to the BIOPEP platform, the highlighted fraction of the peptide Y**AAAPVAVAK** is also characterized as an antioxidant, ACE inhibitor, and pancreatic lipase inhibitor peptide. This sequence was identified in hydrolysates of a *Tenebrio molitor* cuticle protein. According to Zielińska et al. (2018, 2020), this peptide has a strong ability to chelate Fe^2+^ (EC_50_ = 0.108 mg mL^−1^), and its IC_50_ values for inhibition of ACE, lipase, and α‐glucosidase were 8.31, 57.69, and 10.92 µg mL^−1^, respectively.

**FIGURE 4:**
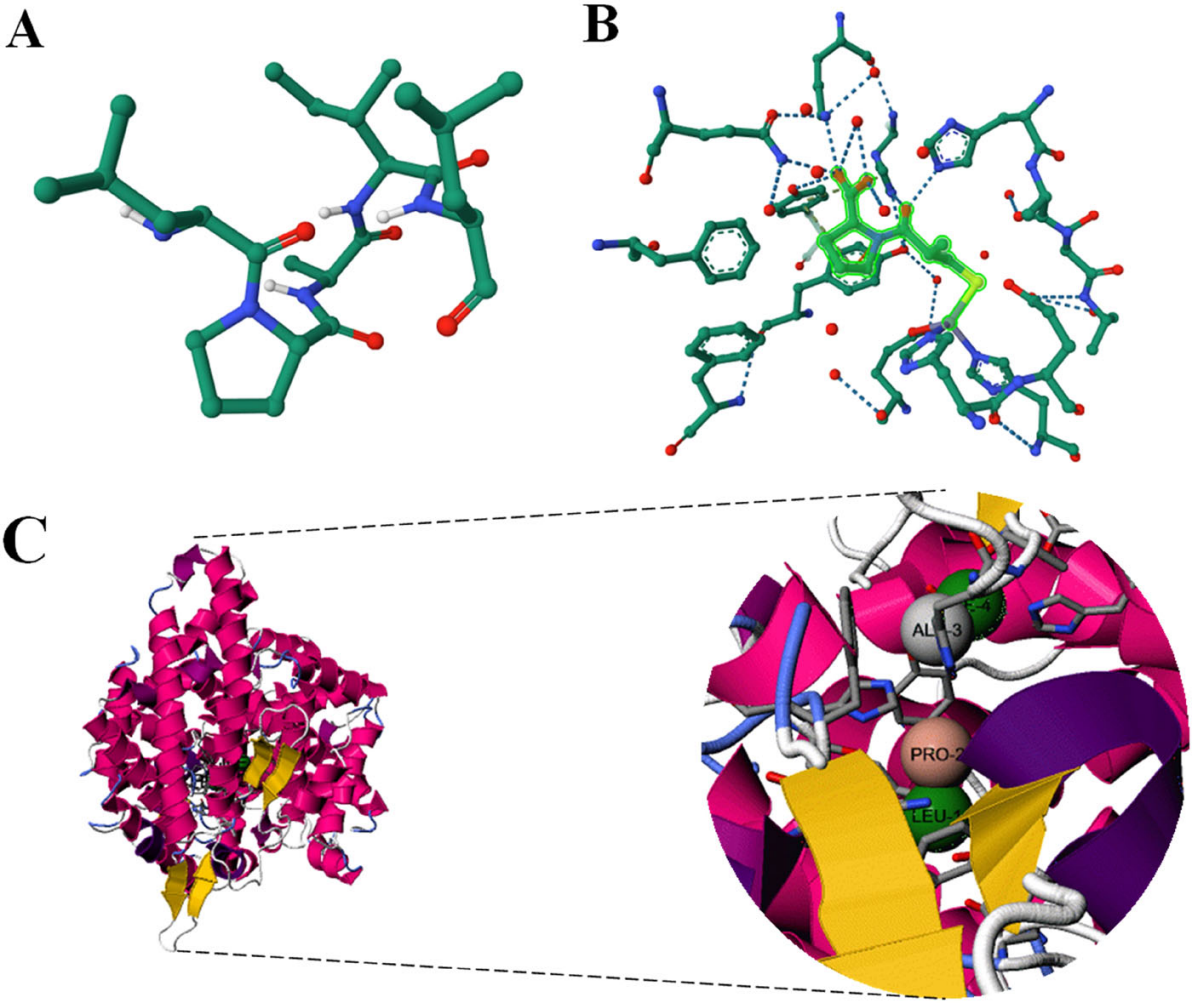
**(A)** LPAIL.pdb visualized on the RCSB PDB server, (B) captopril ligand with interactions with ACE (PDB ID: 1UZF), and (C) molecular docking of the peptide “LPAIL” with the ACE enzyme (1O8A) using PepSite2.

The in silico prediction of the antioxidant activity of the peptides generated four scores higher than 0.5, all indicating potential radical scavenging activity. The FRS scores were 0.65, 0.63, 0.53, and 0.52 for the sequences ADEYDPHPQY, GGYGGY, APLAYGAPVAKY, and APLAYSGGYLH, respectively. For the in silico prediction of antihypertensive peptides, the sequences ITVPLP, ADEYDPHPQY, and APLAYSGGYLH were identified as potential bioactives, with SVM scores of 1.23, 1.31, and 1.37, respectively. The SMILES sequences up to 200 characters were analyzed for their possible macromolecular targets using the SwissTargetPrediction platform. Among the 19 sequences analyzed, the sequence IPAII showed a 57.19% probability of ACE inhibitory activity, while the sequence LPAIL showed probabilities of 66.25% and 61.29% for DPP‐IV and ACE inhibitory activities, respectively. This last sequence had its binding to the ACE enzyme simulated through molecular docking (Figure 4).

The prediction model for the binding of the peptide LPAIL to the surfaces of the target enzyme ACE was generated in the Pepsite2 database, and presents high significance (*p* = 0.000178). The peptide amino acid residues involved in the interaction were Leu1, Pro2, Ala3, and Ile4. As for the receptor residues involved in the inhibition process, these were Trp279, Gln281, His353, Ala354, His383, Glu384, His387, Glu411, Phe457, Phe460, Lys511, Phe512, His513, Tyr520, and Tyr523.

When analyzing the crystallized structure of ACE bound to the inhibitor Captopril (Natesh et al. 2004), it is possible to observe its interaction with the residues Asn66, Ala354, Ser355, Ala356, Trp357, Asp358, Tyr360, Glu384, His387, Phe391, Tyr394, Arg402, Glu403, Gly404, Asn406, Pro407, His410, Glu411, Phe512, Val518, Arg522, Tyr523, Act700, and Zn701 of the ACE enzyme. Notably, among the amino acid residues that interact directly with the ligand captopril, there are 6 amino acid residues that also participate in the interaction of the peptide LPAIL with ACE (Ala354, Glu384, His387, Glu411, Phe512, and Tyr523), which is a strong indication of its antihypertensive properties.

## 4 Conclusion

This study demonstrated that *Tenebrio molitor* is an interesting substrate for obtaining peptides with antioxidant, antidiabetic, and antihypertensive properties. Hydrolysis increased the soluble protein content, facilitating the exploration of its bioactive potential. It was observed that the action profile of the commercial proteases was consistent for hydrolysates derived from both mealworm flour and its protein concentrate, indicating that it is possible to generate mealworm hydrolysates with enhanced properties without the need to produce a protein concentrate. Hydrolysates obtained with flavourzyme presented the best results for most of the bioactivities evaluated. The peptides identified in this sample were composed of residue sequences with potential bioactivity already reported. In silico studies highlighted sequences that were potentially bioactive for specific properties. Furthermore, the comparison of binding sites generated from molecular docking studies with crystallized enzyme structures featuring inhibitor ligands proved to be an interesting strategy for verifying data obtained from in silico studies. This approach not only supports the findings but also enhances the credibility of the predictive models used in peptide characterization. Future research could focus on exploring the bioaccessibility, bioavailability, and toxicity of these peptides, as well as their potential applications in functional foods and nutraceuticals.

## Author Contributions

**Francielle Miranda de Matos**: data curation, formal analysis, writing–original draft preparation, visualization, investigation. **Ruann Janser Soares de Castro**: conceptualization, methodology, resources, writing–review and editing, supervision, project administration, funding acquisition.

## Conflicts of Interest

The authors declare no conflicts of interest.

## Supporting information




**Supplementary Material**: jfds70595‐sup‐0001‐SuppMatt.docx

**Supplementary Material**: jfds70595‐sup‐0002‐SuppMatt.docx

**Supplementary Material**: jfds70595‐sup‐0003‐SuppMatt.docx

**Supplementary Table**: jfds70595‐sup‐0004‐TableS4.docx
