# Supplementary material for: Characterization and Identification of Potential Antioxidant, Antidiabetic, and Antihypertensive Peptides From Hydrolysates of Tenebrio molitor Flour and Its Protein Concentrate
Source: J Food Sci. 2025 Sep 30;90(10):e70595. doi: 10.1111/1750-3841.70595 (PMC12481645; doi:10.1111/1750-3841.70595)
Supplement: Supplementary file 1 — Supplementary Material: jfds70595‐sup‐0001‐SuppMatt.docx [file JFDS-90-0-s003.docx]

**Supplementary material 1**. Analysis of variance (ANOVA) including models, *R*^2^ and probability values for the final reduced models for protein content, percentual of free amino groups, and antioxidant activities of the mealworm’s hydrolysates.

| **Responses** | **Models** | **Equations** | ***F*_calculated_/*_F_*_tabulated_** | ***R*²** | ***p*-value** |
| --- | --- | --- | --- | --- | --- |
|  |  | **Mealworms flour hydrolysate** |  |  |  |
| **Protein content** | Quadratic | $Y=36.72x_{1}+48.20x_{2}+57.06x_{3}-17.00x_{1}x_{3}+16.16x_{2}x_{3}$ | 69.32/5.19 | 0.98 | < 0.0002 |
| **Free amino groups** | Quadratic | $Y=25.68x_{1}+9.22x_{2}+10.03x_{3}+19.45x_{1}x_{2}+27.83x_{1}x_{3}$ | 24.12/5.19 | 0.95 | < 0.002 |
| **ABTS** | Linear | $Y=233.09x_{1}+174.37x_{2}+168.24x_{3}$ | 20.37/4.74 | 0.85 | < 0.002 |
| **DPPH** | Linear | $Y=44.95x_{1}+29.64x_{2}+25.57x_{3}$ | 14.41/4.74 | 0.80 | < 0.004 |
| **FRAP** | Linear | $Y=68.41x_{1}+47.64x_{2}+43.56x_{3}$ | 44.00/4.74 | 0.93 | < 0.0002 |
|  |  | **Mealworms protein concentrate hydrolysate** |  |  |  |
| **Protein content** | Linear | $Y=64.85x_{1}+95.71x_{2}+112.20x_{3}$ | 22.75/4.74 | 0.87 | < 0.0009 |
| **Free amino groups** | Quadratic | $Y=21.59x_{1}+3.76x_{2}+5.70x_{3}+13.56x_{1}x_{2}+10.40x_{1}x_{3}+8.94x_{2}x_{3}$ | 71.20/6.26 | 0.98 | < 0.0006 |
| **ABTS** | Special Cubic | $Y=359.74x_{1}+294.84x_{2}+266.30x_{3}-732.24x_{1}x_{2}x_{3}$ | 21.91/4.76 | 0.92 | < 0.002 |
| **DPPH** | Special Cubic | $Y=10.13x_{1}+5.48x_{2}+5.14x_{3}+ 41.22x_{1}x_{2}x_{3}$ | 14.88/4.76 | 0.88 | < 0.004 |
| **FRAP** | Quadratic | $Y=17.96x_{1}+10.80x_{2}+10.15x_{3}+5.48x_{1}x_{3}$ | 66.37/4.76 | 0.97 | < 0.0001 |

The coded values in model equations represent the independent variables and their interactions: x_1_: Flavourzyme ™; x_2_: Alcalase™; x_3_: Neutrase™.
