# Supplementary material for: Characterization and Identification of Potential Antioxidant, Antidiabetic, and Antihypertensive Peptides From Hydrolysates of Tenebrio molitor Flour and Its Protein Concentrate
Source: J Food Sci. 2025 Sep 30;90(10):e70595. doi: 10.1111/1750-3841.70595 (PMC12481645; doi:10.1111/1750-3841.70595)
Supplement: Supplementary file 2 — Supplementary Material: jfds70595‐sup‐0002‐SuppMatt.docx [file JFDS-90-0-s002.docx]

|  |  |
| --- | --- |
|  |  |
|  |  |

**Supplementary material 2 -** Mixture-contour plots for antioxidant activities of mealworms hydrolysates using a statistical mixture design. The graphics A, B, and C correspond to the flour hydrolysates, evaluated by the ABTS, DPPH, and FRAP methods, respectively. The graphics D, E, and F correspond to the protein concentrate hydrolysates, also evaluated by the ABTS, DPPH, and FRAP methods, respectively.
