# Supplementary material for: Characterization and Identification of Potential Antioxidant, Antidiabetic, and Antihypertensive Peptides From Hydrolysates of Tenebrio molitor Flour and Its Protein Concentrate
Source: J Food Sci. 2025 Sep 30;90(10):e70595. doi: 10.1111/1750-3841.70595 (PMC12481645; doi:10.1111/1750-3841.70595)
Supplement: Supplementary file 3 — Supplementary Material: jfds70595‐sup‐0003‐SuppMatt.docx [file JFDS-90-0-s001.docx]

**Supplementary material 3 -** Validation tests performed to determine the adequacy of the models obtained for antioxidant activities of the hydrolysates from mealworm flour and its protein concentrate.

| **Responses (µmol TEq g^-1^)** | **Independent variables^1^** | | | **Predicted response** | **Experimental response^2^** | **RSD^3^ (%)** |
| --- | --- | --- | --- | --- | --- | --- |
|  | **x_1_** | **x_2_** | **x_3_** |  |  |  |
| **Mealworm flour** | | | | | | |
| **ABTS** | 1 | 0 | 0 | 233.09^a^ | 237.37 ± 7.03^a^ | 1.84 |
| **DPPH** | 1 | 0 | 0 | 44.95^c^ | 44.04 ± 2.99^c^ | -2.02 |
| **FRAP** | 1 | 0 | 0 | 68.41^b^ | 70.49 ± 3.96^b^ | 3.04 |
| **Mealworm protein concentrate** | | | | | | |
| **ABTS** | 1 | 0 | 0 | 359.74^a^ | 359.31 ± 1.67^a^ | -0.12 |
| **DPPH** | 1 | 0 | 0 | 10.13^c^ | 9.71 ± 0.35^c^ | -4.19 |
| **FRAP** | 1 | 0 | 0 | 17.96^b^ | 17.41 ± 0.78^b^ | -3.07 |

x_1_: Flavourzyme ™; x_2_: Alcalase™; x_3_: Neutrase™ represents the coded values for independent variables. ^2^ Results are presented as the mean (n = 3) ± SD and those with different letters are significantly different on the same line (p < 0.05). ^3^RSD (%) = relative standard deviation.
