# Supplementary material for: Characterization and Identification of Potential Antioxidant, Antidiabetic, and Antihypertensive Peptides From Hydrolysates of Tenebrio molitor Flour and Its Protein Concentrate
Source: J Food Sci. 2025 Sep 30;90(10):e70595. doi: 10.1111/1750-3841.70595 (PMC12481645; doi:10.1111/1750-3841.70595)
Supplement: Supplementary file 4 — Supplementary Table: jfds70595‐sup‐0004‐TableS4.docx [file JFDS-90-0-s004.docx]

**Table S4 -** Physiochemical properties of potential bioactive peptides identified from fraction < 3 kDa of mealworm protein concentrate hydrolyzed with Flavourzyme (MPC-H1).

| **Peptide Sequence** | **Molecular Weight** | **Isoelectric Point** | **Net charge at pH 7** | **Estimated water solubility** |
| --- | --- | --- | --- | --- |
| AP**IAY** | 533.63 | 5.85 | 0 | Poor |
| RPQVDLE**TY** | 1120.22 | 4.07 | -1 | Good |
| APLAYSGGY**LH** | 1148.28 | 7.7 | 0 | Poor |
| DVQDG**LT**GDSKN | 1248.26 | 3.58 | -2 | Good |
| Y**AAAPVAVAK** | 960.14 | 9.5 | 1 | Poor |
| **AD**EYDPHPQY | 1234.24 | 3.7 | -3 | Good |
| AMKNFGMK**PE**E | 1281.5 | 7.03 | 0 | Good |
| MKTQ**DP** | 718.82 | 6.53 | 0 | Good |
| **IPA**II | 525.69 | 6.04 | 0 | Poor |
| AP**LAY** | 533.63 | 5.85 | 0 | Poor |
| LPA**IL** | 525.69 | 5.98 | 0 | Poor |
| IEP**IF** | 617.74 | 3.42 | -1 | Poor |
| ITV**PLP** | 638.8 | 5.89 | 0 | Poor |
| GG**YGGY** | 572.58 | 5.8 | 0 | Poor |
| **GL**LEGLD | 715.8 | 2.89 | -2 | Good |
| QAAP**VAV** | 654.76 | 5.71 | 0 | Poor |
| **AAP**VAVAK | 725.89 | 10.05 | 1 | Poor |
| GLIGAP**IA** | 710.87 | 6.02 | 0 | Poor |
| APLAAP**AI** | 722.88 | 5.97 | 0 | Poor |
| APIAAP**IA** | 722.88 | 6.02 | 0 | Poor |
| **AP**IAAPLA | 722.88 | 6.02 | 0 | Poor |
| GLGAPA**LG** | 654.76 | 5.98 | 0 | Poor |
| **GL**IGAPAVA | 767.92 | 6.02 | 0 | Poor |
| **GL**LGAPAVA | 767.92 | 6.02 | 0 | Poor |
| GLIGAPI**AAP** | 879.06 | 5.81 | 0 | Poor |
| AVAAP**VAV**AK | 896.1 | 10.05 | 1 | Poor |
| DIRVSNP**GVR** | 1112.25 | 11 | 1 | Good |
| **AA**YAAPVAHA | 941.05 | 7.74 | 0 | Poor |
| **LG**GNQAVSHY | 1045.12 | 7.74 | 0 | Poor |
| VATYAAAP**VAV** | 1032.2 | 5.93 | 0 | Poor |
| **AA**VAAPVAVAK | 967.17 | 10.05 | 1 | Poor |
| DIRVSNPG**VRF** | 1259.43 | 11 | 1 | Good |
| SLGGNQAVS**HY** | 1132.19 | 7.56 | 0 | Poor |
| **GGY**GSGLGIAR | 1007.11 | 9.85 | 1 | Poor |
| DIQ**DGL**TGDSKN | 1262.29 | 3.58 | -2 | Good |
| APLAYGAPVA**KY** | 1220.43 | 9.55 | 1 | Poor |
| ELHGDSGKGGS**GEP** | 1326.33 | 4.43 | -2 | Good |
| **AP**YAVHAPAVGASHQ | 1475.62 | 7.9 | 0 | Poor |
